# 1,4-Bis[4-(*tert*-butyl­diphenyl­silyl)buta-1,3-diyn­yl]benzene

**DOI:** 10.1107/S160053681000351X

**Published:** 2010-03-13

**Authors:** Damien Thevenet, Reinhard Neier, Helen Stoeckli-Evans

**Affiliations:** a Institute of Chemistry, University of Neuchâtel, rue Emile-Argand 11, 2009 Neuchâtel, Switzerland; b Institute of Physics, University of Neuchâtel, rue Emile-Argand 11, 2009 Neuchâtel, Switzerland

## Abstract

The title centrosymmetric mol­ecule, C_46_H_42_Si_2_, is composed of a central benzene ring with buta-1,3-diynyl chains at positions 1 and 4. These chains are terminated by *tert-*butyl­diphenyl­silyl groups, hence the molecule is dumbbell in shape. The mol­ecules are connected *via* C—H⋯π inter­actions in the structure, so forming an undulating two-dimensional network in the *bc* plane. There is also a weak π–π inter­action involving centrosymmetrically related phenyl rings with a centroid–centroid distance of 3.8359 (11) Å.

## Related literature

For polyynes and acetyl­enic arrays, see: Ginsburg *et al.* (1995 ▶); Siemsen *et al.* (2000 ▶); Brandsma (1988 ▶). For uses and other properties of conjugated carbon–carbon triple bonds, see: Swager (2005 ▶); Tobe & Wakabayashi (2005 ▶); Höger (2005 ▶); Zhou *et al.* (1994 ▶); Maruyama & Kawabata (1990 ▶); Lee *et al.* (2000 ▶). For information on the ‘one-pot’ tandem synthesis – Corey–Fuchs reaction/Negishi coupling, see: Corey & Fuchs (1972 ▶); Desai & McKelvie (1962 ▶); King *et al.* (1977 ▶). For the crystal structure of the trimethyl­silyl analogue, see: Shi Shun *et al.* (2003 ▶). For the synthesis and crystal structure of related compounds, see: Chalifoux *et al.* (2009 ▶); Kim (2009 ▶); West *et al.* (2008 ▶). For a description of the Cambridge Structural Database, see: Allen *et al.* (1987 ▶).
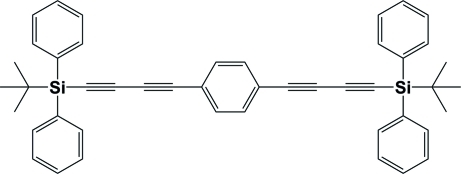


## Experimental

### 

#### Crystal data

C_46_H_42_Si_2_
*M* *_r_* = 650.98
Monoclinic, 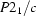

*a* = 8.535 (1) Å
*b* = 17.2060 (14) Å
*c* = 13.4923 (14) Å
β = 104.064 (9)°
*V* = 1922.0 (3) Å^3^
*Z* = 2
Mo *K*α radiation
μ = 0.12 mm^−1^
*T* = 173 K
0.45 × 0.38 × 0.30 mm

#### Data collection

Stoe IPDS-2 diffractometer
Absorption correction: multi-scan (MULscanABS in *PLATON*; Spek, 2009 ▶) *T*_min_ = 0.919, *T*_max_ = 1.184
19707 measured reflections
4403 independent reflections
3260 reflections with *I* > 2σ(*I*)
*R*_int_ = 0.098

#### Refinement

*R*[*F* ^2^ > 2σ(*F* ^2^)] = 0.044
*wR*(*F* ^2^) = 0.104
*S* = 0.96
4403 reflections
220 parameters
H-atom parameters constrained
Δρ_max_ = 0.35 e Å^−3^
Δρ_min_ = −0.29 e Å^−3^

### 

Data collection: *X-AREA* (Stoe & Cie, 2009 ▶); cell refinement: *X-AREA*; data reduction: *X-RED32* (Stoe & Cie, 2009 ▶); program(s) used to solve structure: *SHELXS97* (Sheldrick, 2008 ▶); program(s) used to refine structure: *SHELXL97* (Sheldrick, 2008 ▶); molecular graphics: *Mercury* (Macrae *et al.*, 2006 ▶); software used to prepare material for publication: *SHELXL97* and *PLATON* (Spek, 2009 ▶).

## Supplementary Material

Crystal structure: contains datablocks I, global. DOI: 10.1107/S160053681000351X/fb2180sup1.cif

Structure factors: contains datablocks I. DOI: 10.1107/S160053681000351X/fb2180Isup2.hkl

Additional supplementary materials:  crystallographic information; 3D view; checkCIF report

## Figures and Tables

**Table 1:** C—H⋯π inter­actions (Å, °) *Cg*1 and *Cg*2 are the centroids of the C8–C13 and C14–C19 rings, respectively.

*D*	H	Centroid	C—H	H⋯*Cg*	*D*⋯*Cg*	*D*—H⋯*Cg*
C6	H6	*Cg*2^i^	0.95	2.85	3.7703 (17)	164
C7	H7	*Cg*1^ii^	0.95	2.95	3.8516 (18)	160

Symmetry codes: (i) 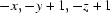; (ii) 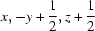.

**Fig. 1.**
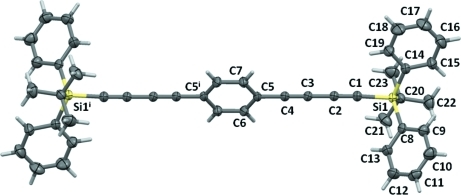
The title molecule with the displacement ellipoids drawn at the 50% probability level [symmetry code: (i) = -x, -y+1, -z+2].

**Fig. 2.**
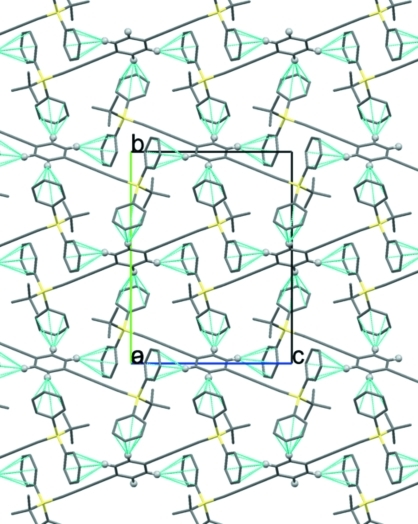
A view along the a-axis of the crystal packing of the title compound. The C—H···π interactions are represented by the H···C contacts shown as dotted cyan lines (see Tab. 1 for details).

**Fig. 3.**
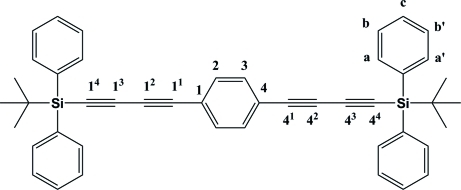
The numbering scheme of the title compound for the interpretation of the NMR spectra.

## supplementary crystallographic information

### Comment

The unique properties of polyynes and acetylenic arrays continue to be of great interest (Ginsburg *et al.*, 1995; Siemsen *et al.*, 2000; Brandsma, 1988). Compounds containing conjugated carbon-carbon triple bonds are important building blocks because they can function as carbon-rich scaffolds when incorporated into organic materials (Swager, 2005; Tobe & Wakabayashi, 2005; Höger, 2005; Zhou *et al.*, 1994). Consequently, research into the synthesis of well-defined polyynes continues to expand. The efficiency of the energy and electron transfer processes in polyyne-bridged porphyrin systems (Maruyama & Kawabata, 1990) and bis(benzocrown ether)s (Lee *et al.*, 2000) have been examined for their potential use as molecular wires and chemosensors.

The title compound was designed as a spacer-unit in linked materials for the creation of structured, discotic mesophases. It was synthesized from *tert*-Butyl(4,4-dibromobut-3-en-1-ynyl)diphenylsilane using a "one-pot" tandem synthesis, consisting of a Corey-Fuchs reaction (Corey & Fuchs, 1972; Desai & McKelvie, 1962) and a Negishi coupling reaction (King *et al.*, 1977). The synthesis and crystal structure of the trimethylsilyl analogue has been described by (Shi Shun *et al.*, 2003), and for some other related compounds by Chalifoux *et al.*, 2009; Kim, 2009; West *et al.* (2008).

The title molecule is shown in Fig. 1. The bond lengths are normal (Allen *et al.*, 1987) and the geometrical parameters are similar to those in the centrosymmetric trimethylsilyl analogue mentioned above (Shi Shun *et al.*, 2003). The title molecule consists of a central benzene ring to which are attached buta-1,3-diynyl chains in positions 1 and 4. These chains are terminated with *tert*-butyldiphenylsilyl groups. The molecule is essentially linear and shaped like a dumbbell. The centers of the benzene rings are situated on crystallographic centers of symmetry, therefore the molecule has symmetry 1.

In the crystal of the title compound symmetry related molecules are connected via C—H···π interactions, involving the H-atoms of the central aromatic ring and the silyl phenyl rings, giving rise to the formation of an undulating two-dimensional network in the *bc* plane (Tab. 1 and Fig. 2). Centrosymmetrically related phenyl rings (C14 - C19), are involved in a weak π–π interaction with a centroid-to-centroid distance [Cg1···Cg1^i^, symmetry code: (i) = 1-x, 1-y, 1-z] of 3.836 (1) Å.

### Experimental

The synthesis of the title compound was carried out under a nitrogen atmosphere. To a solution of *tert*-butyl(4,4-dibromobut-3-en-1-ynyl)diphenylsilane (2.64 mmol in 5.0 ml of dry tetrahydrofuran) was added N-butyl lithium (3.63 ml of 1.6 M in hexane; 5.81 mmol) at 193 K. The mixture was stirred at 193 K to 233 K for 2 h. Anhydrous ZnCl_2_ (5.28 mmol dissolved in 5.0 ml of tetrahydrofuran) was then added and the mixture was stirred at 233 K to 293 K for 1 h. Subsequently 1,4-diiodobenzene (0.88 mmol dissolved in 5 ml of dimethylformamide) and Pd(dppf)Cl_2_ [dppf = 1,1'-bis(diphenylphosphino)ferrocene] (0.17 mmol dissolved in 5 ml of CH_2_Cl_2_) were added and the mixture stirred at 353 K for 24 h. The reaction mixture was then filtered over Celite (a diatomaceous earth, which is a naturally occurring, soft, siliceous sedimentray rock, used for filtration purposes) and concentrated. The crude product was purified by column chromatography (silica gel, petroleum ether:CH_2_Cl_2_ (9:1)). Colourless rod-like crystals (average size 0.8 × 0.4 × 0.3 mm) of the title compound were grown by slow evaporation of a concentrated solution in hexane at 277 K.

^1^H NMR, 400 MHz (CDCl_3_) δ 7.80 (m, 8H, H_a,a'_), 7.51 (s, 4H, H_2,3,5,6_), 7.46-7.38 (m, 12H, H_b,b',c_), 1.14 (s, 18H, C(CH_3_)_3_)) ; ^13^C NMR, 100 MHz (CDCl_3_) δ 135.7 (C_a,a'_), 132.9 (C_2,3,5,6_), 132.5 (C_ar_-Si), 129.9 (C_c_), 128.0 (C_b,b'_), 122.5 (C_1,4_), (91.3, 80.0, 77.3) (C_1_^2^,_1_^3^,_1_^4^, _4_^2^,_4_^3^,_4_^4^), 76.34 (C_1_^1^,_4_^1^), 27.2 (C(CH_3_)_3_), 19.1 (C(CH_3_)_3_); HRMS (ESI, +): [M+Na]^+^ = 673.27201. The numbering scheme for the interpretation of the NMR spectra is given in Fig. 3.

### Refinement

The H-atoms could all be located in difference electron-density maps. In the final cycles of refinement they were included in calculated positions and treated as riding atoms: C—H = 0.95 Å for H-aryl and 0.98 Å for methyl H-atoms, with U_iso_(H) = k × U_eq_(parent C-atom), where k = 1.2 for aryl and k = 1.5 for methyl H-atoms.

### Crystal data

**Table:**

C_46_H_42_Si_2_	*F*(000) = 692
*M**_r_* = 650.98	*D*_x_ = 1.125 Mg m^−^^3^
Monoclinic, *P*2_1_/*c*	Mo *K*α radiation, λ = 0.71073 Å
Hall symbol: -P 2ybc	Cell parameters from 13367 reflections
*a* = 8.535 (1) Å	θ = 2.0–29.6°
*b* = 17.2060 (14) Å	µ = 0.12 mm^−^^1^
*c* = 13.4923 (14) Å	*T* = 173 K
β = 104.064 (9)°	Block, colourless
*V* = 1922.0 (3) Å^3^	0.45 × 0.38 × 0.30 mm
*Z* = 2	

### Data collection

**Table:**

Stoe IPDS-2 diffractometer	4403 independent reflections
Radiation source: fine-focus sealed tube	3260 reflections with *I* > 2σ(*I*)
graphite	*R*_int_ = 0.098
φ and ω scans	θ_max_ = 27.5°, θ_min_ = 2.0°
Absorption correction: multi-scan (MULscanABS in *PLATON*; Spek, 2009)	*h* = −11→11
*T*_min_ = 0.919, *T*_max_ = 1.184	*k* = −22→22
19707 measured reflections	*l* = −16→17

### Refinement

**Table:**

Refinement on *F*^2^	Primary atom site location: structure-invariant direct methods
Least-squares matrix: full	Secondary atom site location: difference Fourier map
*R*[*F*^2^ > 2σ(*F*^2^)] = 0.044	Hydrogen site location: difference Fourier map
*wR*(*F*^2^) = 0.104	H-atom parameters constrained
*S* = 0.96	*w* = 1/[σ^2^(*F*_o_^2^) + (0.0586*P*)^2^] where *P* = (*F*_o_^2^ + 2*F*_c_^2^)/3
4403 reflections	(Δ/σ)_max_ = 0.001
220 parameters	Δρ_max_ = 0.35 e Å^−^^3^
0 restraints	Δρ_min_ = −0.29 e Å^−^^3^
81 constraints	

### Special details

**Table:**

Geometry. Bond distances, angles etc. have been calculated using the rounded fractional coordinates. All su's are estimated from the variances of the (full) variance-covariance matrix. The cell esds are taken into account in the estimation of distances, angles and torsion angles
Refinement. Refinement of *F*^2^ against ALL reflections. The weighted *R*-factor wR and goodness of fit *S* are based on *F*^2^, conventional *R*-factors *R* are based on *F*, with *F* set to zero for negative *F*^2^. The threshold expression of *F*^2^ > σ(*F*^2^) is used only for calculating *R*-factors(gt) etc. and is not relevant to the choice of reflections for refinement. *R*-factors based on *F*^2^ are statistically about twice as large as those based on *F*, and *R*- factors based on ALL data will be even larger.

### Fractional atomic coordinates and isotropic or equivalent isotropic displacement parameters (Å^2^)

**Table:**

	*x*	*y*	*z*	*U*_iso_*/*U*_eq_	
Si1	0.21188 (5)	0.33985 (2)	0.45574 (3)	0.0274 (1)	
C1	0.17432 (18)	0.37157 (8)	0.57829 (12)	0.0319 (4)	
C2	0.15087 (18)	0.39564 (8)	0.65764 (11)	0.0304 (4)	
C3	0.12243 (18)	0.42333 (8)	0.74707 (11)	0.0313 (4)	
C4	0.09221 (18)	0.44715 (8)	0.82412 (11)	0.0312 (4)	
C5	0.04697 (17)	0.47453 (8)	0.91335 (11)	0.0284 (4)	
C6	−0.09906 (19)	0.51441 (9)	0.90339 (11)	0.0330 (4)	
C7	0.14561 (19)	0.46019 (9)	1.01075 (11)	0.0331 (5)	
C8	0.35951 (17)	0.25716 (8)	0.48565 (11)	0.0305 (4)	
C9	0.4176 (2)	0.21781 (10)	0.41082 (14)	0.0442 (5)	
C10	0.5271 (2)	0.15713 (11)	0.43600 (17)	0.0545 (6)	
C11	0.5821 (2)	0.13405 (11)	0.53583 (19)	0.0556 (7)	
C12	0.5256 (2)	0.17083 (11)	0.61040 (16)	0.0559 (7)	
C13	0.4161 (2)	0.23146 (10)	0.58596 (13)	0.0397 (5)	
C14	0.29838 (17)	0.42645 (8)	0.40325 (11)	0.0304 (4)	
C15	0.2355 (2)	0.50019 (9)	0.41244 (14)	0.0423 (5)	
C16	0.2954 (2)	0.56598 (10)	0.37522 (15)	0.0478 (6)	
C17	0.4212 (2)	0.55952 (10)	0.32833 (14)	0.0458 (6)	
C18	0.4861 (2)	0.48762 (11)	0.31796 (16)	0.0504 (6)	
C19	0.4256 (2)	0.42169 (10)	0.35525 (14)	0.0420 (5)	
C20	0.00954 (18)	0.30897 (9)	0.37155 (13)	0.0346 (4)	
C21	−0.0513 (2)	0.23744 (11)	0.41817 (17)	0.0535 (7)	
C22	0.0254 (2)	0.28984 (12)	0.26356 (14)	0.0521 (6)	
C23	−0.1143 (2)	0.37500 (11)	0.36436 (16)	0.0518 (6)	
H6	−0.16650	0.52410	0.83740	0.0400*	
H7	0.24460	0.43300	1.01790	0.0400*	
H9	0.38100	0.23300	0.34130	0.0530*	
H10	0.56440	0.13140	0.38380	0.0650*	
H11	0.65830	0.09300	0.55310	0.0670*	
H12	0.56190	0.15460	0.67950	0.0670*	
H13	0.37870	0.25610	0.63890	0.0480*	
H15	0.14930	0.50540	0.44510	0.0510*	
H16	0.24970	0.61550	0.38200	0.0570*	
H17	0.46320	0.60460	0.30320	0.0550*	
H18	0.57240	0.48300	0.28520	0.0610*	
H19	0.47180	0.37240	0.34790	0.0500*	
H21A	−0.06650	0.25060	0.48590	0.0800*	
H21B	0.02800	0.19540	0.42450	0.0800*	
H21C	−0.15440	0.22050	0.37400	0.0800*	
H22A	0.06540	0.33560	0.23410	0.0780*	
H22B	−0.08040	0.27500	0.22090	0.0780*	
H22C	0.10140	0.24670	0.26660	0.0780*	
H23A	−0.12390	0.38880	0.43310	0.0780*	
H23B	−0.21950	0.35780	0.32320	0.0780*	
H23C	−0.07830	0.42050	0.33220	0.0780*	

### Atomic displacement parameters (Å^2^)

**Table:**

	*U*^11^	*U*^22^	*U*^33^	*U*^12^	*U*^13^	*U*^23^
Si1	0.0368 (2)	0.0266 (2)	0.0211 (2)	0.0044 (2)	0.0119 (2)	−0.0017 (2)
C1	0.0410 (8)	0.0295 (7)	0.0284 (8)	0.0031 (6)	0.0144 (6)	−0.0001 (6)
C2	0.0400 (8)	0.0268 (7)	0.0270 (8)	0.0025 (6)	0.0131 (6)	0.0007 (5)
C3	0.0434 (8)	0.0276 (7)	0.0251 (7)	0.0022 (6)	0.0128 (6)	0.0007 (5)
C4	0.0443 (8)	0.0256 (7)	0.0258 (8)	0.0017 (6)	0.0126 (6)	0.0002 (5)
C5	0.0414 (8)	0.0238 (6)	0.0222 (7)	−0.0018 (5)	0.0123 (6)	−0.0034 (5)
C6	0.0428 (8)	0.0344 (8)	0.0205 (7)	0.0048 (6)	0.0053 (6)	−0.0006 (5)
C7	0.0385 (8)	0.0343 (8)	0.0274 (8)	0.0069 (6)	0.0099 (6)	−0.0016 (6)
C8	0.0347 (7)	0.0297 (7)	0.0286 (8)	0.0012 (6)	0.0106 (6)	−0.0017 (6)
C9	0.0543 (10)	0.0447 (9)	0.0381 (9)	0.0140 (8)	0.0200 (8)	−0.0009 (7)
C10	0.0552 (10)	0.0494 (10)	0.0658 (13)	0.0164 (9)	0.0279 (10)	−0.0062 (9)
C11	0.0427 (9)	0.0473 (10)	0.0738 (15)	0.0183 (8)	0.0083 (9)	0.0025 (9)
C12	0.0556 (11)	0.0562 (12)	0.0482 (11)	0.0151 (9)	−0.0023 (9)	0.0086 (9)
C13	0.0449 (8)	0.0413 (9)	0.0309 (8)	0.0075 (7)	0.0052 (7)	0.0000 (7)
C14	0.0377 (8)	0.0326 (7)	0.0206 (7)	0.0008 (6)	0.0065 (6)	−0.0013 (5)
C15	0.0587 (10)	0.0329 (8)	0.0408 (10)	0.0019 (7)	0.0230 (8)	−0.0018 (7)
C16	0.0661 (11)	0.0312 (8)	0.0468 (11)	−0.0019 (8)	0.0151 (9)	−0.0006 (7)
C17	0.0515 (10)	0.0422 (9)	0.0400 (10)	−0.0132 (8)	0.0040 (8)	0.0058 (7)
C18	0.0444 (9)	0.0535 (11)	0.0583 (12)	−0.0030 (8)	0.0219 (9)	0.0091 (9)
C19	0.0415 (8)	0.0396 (9)	0.0494 (11)	0.0023 (7)	0.0195 (8)	0.0043 (7)
C20	0.0388 (8)	0.0296 (7)	0.0348 (8)	0.0038 (6)	0.0079 (6)	−0.0041 (6)
C21	0.0524 (10)	0.0420 (10)	0.0671 (14)	−0.0065 (8)	0.0165 (10)	0.0016 (9)
C22	0.0584 (11)	0.0587 (11)	0.0357 (10)	−0.0017 (9)	0.0049 (8)	−0.0165 (8)
C23	0.0429 (9)	0.0461 (10)	0.0598 (13)	0.0130 (8)	−0.0001 (9)	−0.0124 (9)

### Geometric parameters (Å, °)

**Table:**

Si1—C1	1.8418 (16)	C20—C22	1.532 (2)
Si1—C8	1.8782 (15)	C20—C23	1.539 (2)
Si1—C14	1.8764 (15)	C6—H6	0.9500
Si1—C20	1.8978 (17)	C7—H7	0.9500
C1—C2	1.209 (2)	C9—H9	0.9500
C2—C3	1.373 (2)	C10—H10	0.9500
C3—C4	1.202 (2)	C11—H11	0.9500
C4—C5	1.431 (2)	C12—H12	0.9500
C5—C6	1.400 (2)	C13—H13	0.9500
C5—C7	1.400 (2)	C15—H15	0.9500
C6—C7^i^	1.384 (2)	C16—H16	0.9500
C8—C9	1.402 (2)	C17—H17	0.9500
C8—C13	1.394 (2)	C18—H18	0.9500
C9—C10	1.387 (3)	C19—H19	0.9500
C10—C11	1.373 (3)	C21—H21A	0.9800
C11—C12	1.372 (3)	C21—H21B	0.9800
C12—C13	1.386 (3)	C21—H21C	0.9800
C14—C15	1.395 (2)	C22—H22A	0.9800
C14—C19	1.395 (2)	C22—H22B	0.9800
C15—C16	1.386 (2)	C22—H22C	0.9800
C16—C17	1.377 (3)	C23—H23A	0.9800
C17—C18	1.376 (3)	C23—H23B	0.9800
C18—C19	1.390 (3)	C23—H23C	0.9800
C20—C21	1.529 (3)		
			
C1—Si1—C8	106.60 (7)	C6^i^—C7—H7	120.00
C1—Si1—C14	105.89 (6)	C8—C9—H9	119.00
C1—Si1—C20	106.78 (7)	C10—C9—H9	119.00
C8—Si1—C14	112.19 (7)	C9—C10—H10	120.00
C8—Si1—C20	112.45 (7)	C11—C10—H10	120.00
C14—Si1—C20	112.38 (7)	C10—C11—H11	120.00
Si1—C1—C2	177.19 (13)	C12—C11—H11	120.00
C1—C2—C3	179.29 (17)	C11—C12—H12	120.00
C2—C3—C4	177.85 (17)	C13—C12—H12	120.00
C3—C4—C5	176.80 (17)	C8—C13—H13	119.00
C4—C5—C6	119.77 (13)	C12—C13—H13	119.00
C4—C5—C7	120.55 (14)	C14—C15—H15	119.00
C6—C5—C7	119.65 (14)	C16—C15—H15	119.00
C5—C6—C7^i^	120.29 (14)	C15—C16—H16	120.00
C5—C7—C6^i^	120.06 (15)	C17—C16—H16	120.00
Si1—C8—C9	123.17 (12)	C16—C17—H17	120.00
Si1—C8—C13	120.33 (12)	C18—C17—H17	120.00
C9—C8—C13	116.50 (14)	C17—C18—H18	120.00
C8—C9—C10	121.47 (17)	C19—C18—H18	120.00
C9—C10—C11	120.49 (18)	C14—C19—H19	119.00
C10—C11—C12	119.27 (18)	C18—C19—H19	119.00
C11—C12—C13	120.65 (19)	C20—C21—H21A	109.00
C8—C13—C12	121.63 (16)	C20—C21—H21B	109.00
Si1—C14—C15	119.58 (12)	C20—C21—H21C	110.00
Si1—C14—C19	123.40 (11)	H21A—C21—H21B	110.00
C15—C14—C19	117.02 (14)	H21A—C21—H21C	109.00
C14—C15—C16	121.81 (16)	H21B—C21—H21C	109.00
C15—C16—C17	119.88 (16)	C20—C22—H22A	109.00
C16—C17—C18	119.82 (16)	C20—C22—H22B	109.00
C17—C18—C19	120.17 (17)	C20—C22—H22C	109.00
C14—C19—C18	121.29 (16)	H22A—C22—H22B	109.00
Si1—C20—C21	109.25 (12)	H22A—C22—H22C	109.00
Si1—C20—C22	110.59 (11)	H22B—C22—H22C	109.00
Si1—C20—C23	110.02 (11)	C20—C23—H23A	109.00
C21—C20—C22	109.62 (15)	C20—C23—H23B	109.00
C21—C20—C23	108.87 (14)	C20—C23—H23C	109.00
C22—C20—C23	108.48 (15)	H23A—C23—H23B	109.00
C5—C6—H6	120.00	H23A—C23—H23C	109.00
C7^i^—C6—H6	120.00	H23B—C23—H23C	109.00
C5—C7—H7	120.00		
			
C1—Si1—C8—C9	−179.84 (13)	C4—C5—C6—C7^i^	178.71 (14)
C1—Si1—C8—C13	0.69 (15)	C7—C5—C6—C7^i^	0.2 (2)
C14—Si1—C8—C9	−64.36 (15)	C4—C5—C7—C6^i^	−178.70 (14)
C14—Si1—C8—C13	116.16 (13)	C6—C5—C7—C6^i^	−0.2 (2)
C20—Si1—C8—C9	63.47 (15)	C5—C6—C7^i^—C5^i^	−0.2 (2)
C20—Si1—C8—C13	−116.00 (13)	Si1—C8—C9—C10	179.77 (13)
C1—Si1—C14—C15	−40.17 (15)	C13—C8—C9—C10	−0.7 (2)
C1—Si1—C14—C19	139.24 (14)	Si1—C8—C13—C12	−179.70 (13)
C8—Si1—C14—C15	−156.07 (13)	C9—C8—C13—C12	0.8 (2)
C8—Si1—C14—C19	23.34 (16)	C8—C9—C10—C11	−0.2 (3)
C20—Si1—C14—C15	76.05 (14)	C9—C10—C11—C12	1.1 (3)
C20—Si1—C14—C19	−104.54 (14)	C10—C11—C12—C13	−1.0 (3)
C1—Si1—C20—C21	−65.38 (13)	C11—C12—C13—C8	0.1 (3)
C1—Si1—C20—C22	173.89 (12)	Si1—C14—C15—C16	179.82 (14)
C1—Si1—C20—C23	54.08 (13)	C19—C14—C15—C16	0.4 (3)
C8—Si1—C20—C21	51.20 (13)	Si1—C14—C19—C18	−179.71 (14)
C8—Si1—C20—C22	−69.54 (13)	C15—C14—C19—C18	−0.3 (3)
C8—Si1—C20—C23	170.66 (11)	C14—C15—C16—C17	−0.5 (3)
C14—Si1—C20—C21	178.93 (11)	C15—C16—C17—C18	0.5 (3)
C14—Si1—C20—C22	58.20 (13)	C16—C17—C18—C19	−0.4 (3)
C14—Si1—C20—C23	−61.61 (14)	C17—C18—C19—C14	0.3 (3)

Symmetry codes: (i) −*x*, −*y*+1, −*z*+2.

### Table 1 C—H···π interactions (Å, °)

Cg1 and Cg2 are the centroids of the C8–C13 and C14–C19 rings, respectively.

**Table:**

*D*	H	Centroid	C—H	H···Cg	*D*···Cg	*D*—H···Cg
C6	H6	Cg2^i^	0.95	2.85	3.7703 (17)	164
C7	H7	Cg1^ii^	0.95	2.95	3.8516 (18)	160

Symmetry codes (i) = -x, -y+1, -z+1; (ii) x, -y+1/2, z+1/2.
